# Promoting Supportive and Respectful Maternity Care in Public Health Facilities in Sindh, Pakistan: A Theory-Informed Health System Intervention

**DOI:** 10.9745/GHSP-D-22-00513

**Published:** 2023-06-21

**Authors:** Bilal Iqbal Avan, Waqas Hameed, Bushra Khan, Muhammad Asim, Sarah Saleem, Sameen Siddiqi

**Affiliations:** a Department of Population Health, London School of Hygiene & Tropical Medicine, London, United Kingdom.; b Department of Community Health Sciences, Aga Khan University, Karachi, Pakistan.; c Department of Psychology, University of Karachi, Karachi, Pakistan.

## Abstract

**Background:**

Disrespect, abuse, discrimination, and lack of emotional support characterize intrapartum care in the health systems of many low- and middle-income countries. Although the World Health Organization (WHO) provides frameworks and guidelines to address this issue, no operational model exists that effectively incorporates WHO intrapartum care guidelines into routine public health services. We aimed to develop and pilot-test a theory-driven, service-delivery intervention package linking dignified care with perinatal mental health to promote psychosocially supportive and respectful maternity care (S-RMC) in public health facilities in Sindh, Pakistan.

**Methods:**

Using a mixed-method, pre-post design, the study was implemented in 6 secondary-level public health facilities in 2 rural districts of Southern Sindh, Pakistan. Its development was guided by the COM-B framework and informed by a literature review, formative research, and consultative sessions with implementers. The intervention was implemented in March–September 2021 and compared women’s experiences of S-RMC during childbirth at baseline (n=313) and endline (n=314). We used descriptive statistics and linear regression techniques for analysis.

**Results:**

A substantial reduction was observed in the cumulative level of overall mistreatment from baseline to endline, yielding a relative change of 50% (*P*<.001). Similar change was evident across different types of mistreatment: physical abuse (75%), verbal abuse (72%), ineffective communication (60%), nonconfidential care (78%), health system conditions and constraints (25%), noninclusive care (28%), lack of supportive care (52%), and stigma and discrimination (82%). Furthermore, we observed a significant reduction in the proportion of women experiencing symptoms of anxiety and depression before and after the intervention.

**Conclusion:**

This intervention built the capacity of maternity teams while improving accountability, health information systems, and governance measures. Given its promise to promote supportive and respectful childbirth in public health facilities, a large-scale effectiveness evaluation across diverse settings is warranted.

## Abstract (precis)

Mistreatment during childbirth is prevalent in many LMICs. The authors piloted a health system intervention to build maternity teams’ capacity to provide inclusive, supportive, and respectful maternity care to women during childbirth.

## Teaser

**Key Findings:**

- We piloted an intervention to improve supportive, respectful maternal care by addressing factors at the individual, provider, and health system levels.
- After the intervention, women reported experiencing a reduction in overall mistreatment, physical and verbal abuse, and stigma and discrimination, as well as a decrease in postpartum anxiety and depressive symptoms.
- Our pioneering health system intervention built the capacity of maternity teams and improved accountability, information systems, and governance measures in facilities, resulting in provision of more inclusive, supportive, and respectful care during childbirth and psychosocial support to women.

**Key Implication:**

- Contextual adaptation and large-scale evaluation of the intervention should be conducted in diverse health settings.

## INTRODUCTION

There is international consensus that disrespectful and abusive care violates women’s right to be treated with dignity, integrity, and respect.^1^ Yet, in most low- and middle-income countries, women face denigrating attitudes from service providers, lack of emotional support, and discriminatory treatment during intrapartum care at health facilities.^2^ The likely consequences of such lamentable experiences include negative birthing outcomes, poor perinatal mental health, and adverse effects on future health-seeking behaviors.^3^ In addition to the lack of respectful care, women struggle with socioeconomic and health-related vulnerabilities that influence their experience of childbirth and its outcomes and predispose them to further discriminatory care^2^^,^^4^ with a likelihood of increased suffering.

In 2014, the World Health Organization (WHO) recognized dignified and respectful care as a fundamental right of every woman.^1^ Their revised framework for quality maternity and newborn health care,^5^ along with evidence-based policy recommendations, promotes positive experiences of intrapartum care.^6^ WHO’s framework and guidelines call for integrated health services that respect women’s dignity during maternity care while also fulfilling their psychosocial needs. With a psychologically safe environment in mind, WHO’s Mental Health Gap Action Programme (mhGAP) has apt materials for addressing women’s psychosocial needs during intrapartum care.

However, despite its critical need to be used, we know of no evidence-based operational model that systematically and effectively incorporates WHO’s guidelines in routine facility-based maternity care.^7^ Interventions to date have been only partially effective (evidence rated with low certainty)^7^ and hamstrung by limited acceptability and feasibility in low-resource settings. Having failed to embed and integrate sufficiently in some health systems, the sustainability of their effects has been questioned.^7^ Furthermore, although providing supportive care is part of WHO’s recommendations for respectful maternity care (RMC), most intervention models and programmatic efforts have focused on reducing disrespect and abuse, with supportive care receiving scant attention.^7^

We conducted early-phase implementation research to develop, implement, and pilot-test a psychosocially supportive and respectful maternity care (S-RMC) intervention package.^8^ S-RMC operationally refers to care that, without any discrimination, safeguards a pregnant woman’s dignity, privacy, and confidentiality, shares information with her to ensure shared decision-making, and is responsive to her psychosocial needs through care coordination. Our approach was consensus-driven and participatory: S-RMC relied on working closely with service providers and health administrators and was informed by contextual evidence.^9^ This article examines the effect of a pilot, theory-driven health system intervention on women’s experiences of mistreatment during childbirth.

S-RMC refers to care that, without any discrimination, safeguards a pregnant woman’s dignity, privacy, and confidentiality, shares information with her to ensure shared decision-making, and is responsive to her psychosocial needs through care coordination.

## THE S-RMC INTERVENTION

### Theoretical Underpinnings

We used the COM-B (capability, opportunity, motivation, and behavior) framework to develop the S-RMC intervention.^10^ The COM-B is a well-studied model used to develop an in-depth understanding of the behavior change of health care staff.^11^^,^^12^ Its premise, a particular behavior, is a function of both psychological and physical abilities, along with the social and physical opportunity to perform that behavior more than other competing behavior possibilities. Our behavior of interest was positive change in the provision of S-RMC. The intervention aimed to improve the capability of maternity care staff to provide inclusive, supportive, and respectful care by inculcating knowledge and skills through training and supportive supervision. Opportunity to enact such behaviors was created by systemic changes within health care facilities and by instilling an ethos of collaborative care. Health care staff were motivated and their attitudes were improved by reinforcing the benefits of inclusive care for individuals, health systems, and communities and by providing psychosocial support among coworkers.^13^ Our hypothesis was that by building maternity staff’s capacity to provide dignified and collaborative care, improving women’s and companions’ orientation to rights to respectful care, and embedding review and accountability mechanisms in health facilities, maternity service providers would be enabled to provide S-RMC (Figure).^8^

**FIGURE:**
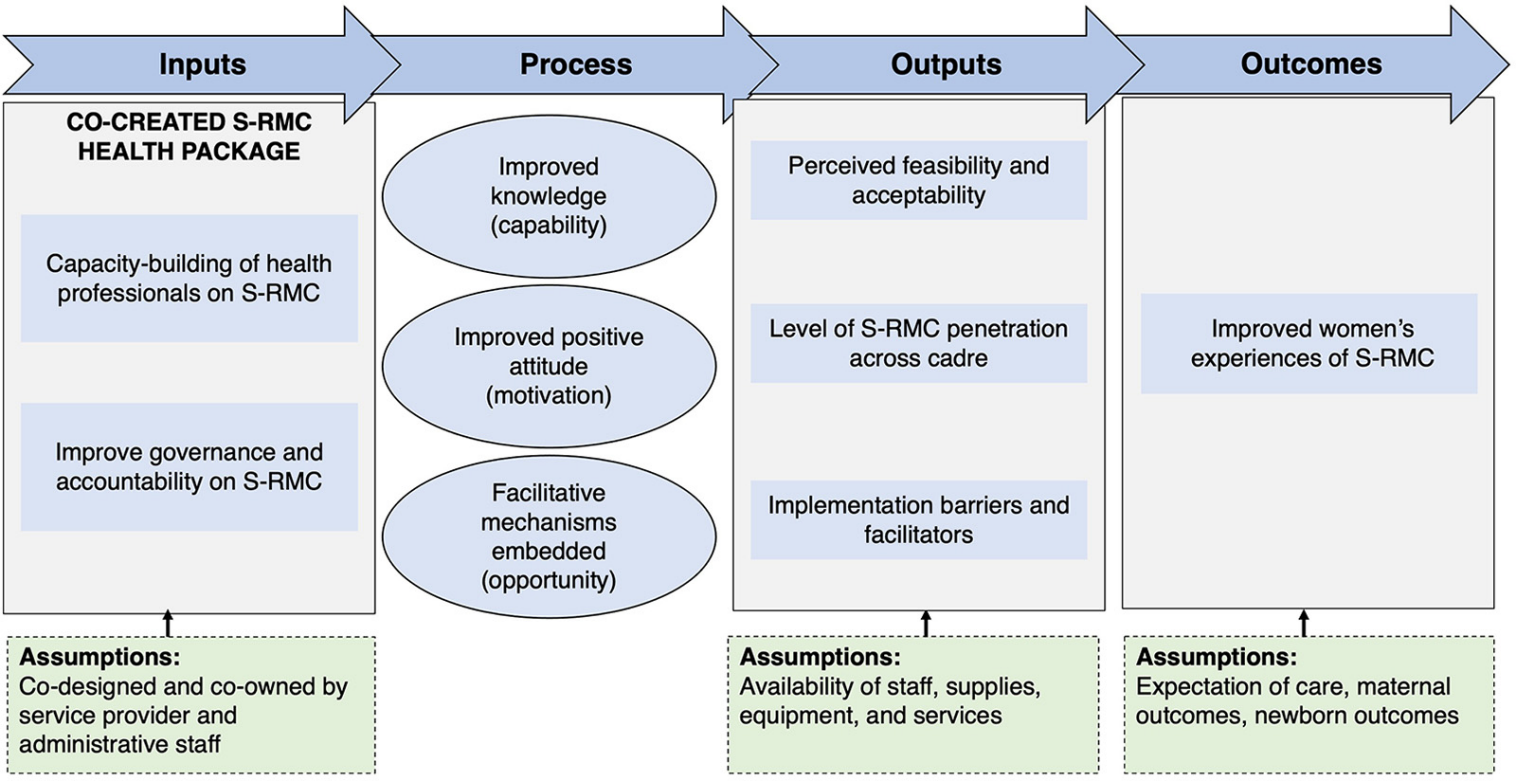
Logic Model of Supportive and Respectful Maternity Care Abbreviation: S-RMC, supportive and respectful maternity care.

### Intervention Description

The S-RMC intervention was a theory-driven, cocreated service delivery package that was developed using a human-centered design approach. Details are included in earlier studies.^8^^,^^14^ Its broad components included capacity-building of maternity teams and systemic changes for improvements in governance and accountability within public health facilities. Uniquely, we integrated psychosocial support in routine maternity care to address the psychological and social needs of birthing women and their companions. Maternity staff were equipped with job aids, including an S-RMC training handbook, to assist them in reassuring women about their rights and responsibilities, referencing essential S-RMC behaviors, and providing psychosocial support. The technical content of the S-RMC training handbook was based on 2 integrated modules linking respectful care with perinatal mental health: WHO’s intrapartum care guidelines^6^ and mhGAP training and implementation guidelines.^15^ The third module focuses on the S-RMC strategy and guidance on its implementation at a health facility level.

The logic model details how the S-RMC intervention systematically modifies how the maternity staff treats women (Figure). All maternity team members (clinical staff, nonclinical staff, and administrators) received training from the research team in team-based care, supportive and respectful care, ethical and rights-based care, and psychosocial care. During this training, the research team devised a customized strategy to operationalize S-RMC in routine intrapartum care. After the training, S-RMC activities and processes were embedded within health facilities, including S-RMC record-keeping (e.g., screening for vulnerability at admissions), benchmarking respectful care and psychosocial support based on screening assessments, and introducing a woman’s complaint system and exit interviews to gather S-RMC experiences. In terms of administrative and information system activities, all S-RMC-related data are consolidated monthly for discussion and remedial actions in monthly performance review meetings for continual quality improvement. The best performance by a team member is awarded S-RMC carer of the month, and on-the-job training and guidance are offered for continuous professional development. A mental health first aider, who is a senior clinical member of the maternity team, is responsible for implementing these activities and processes. Maternity staff are equipped with job aids (e.g., record-keeping registers to screen for patient vulnerability, exit interview forms, monthly report format, and S-RMC posters). Finally, supportive supervision helped to embed essential S-RMC behaviors and helped staff take steps to provide psychosocial support. During the first month of the pilot test, the research team conducted supportive supervision visits weekly. In subsequent months, visits were changed to a monthly basis. The supportive supervision role was then given to the maternity team leads and facility managers.

## METHODS

### Study Design

We used a pre-/post-survey design, with a comparison group to pilot-test an intervention package aiming to promote a culture of psychosocially S-RMC in public health facilities.^8^ The assessment compared women’s experiences of mistreatment during childbirth before and after the intervention. The pre-intervention assessment was conducted from September to December 2020, followed by a 6-month intervention period that began in March 2021 and an endline assessment from September to December 2021.

### Study Settings

The study was implemented in the Thatta and Sujawal districts of Sindh province, Pakistan. These contiguous districts, which were selected in consultation with government and local stakeholders, are predominately rural (82%) and 100 km from the nearest large city, Karachi. Each district has a population of approximately 0.9 million,^16^ low literacy (18%), and nearly half of its population in the poorest wealth quintile relative to Sindh province.^17^ The indicators of maternal, newborn, and child health are relatively poor.^18^ The intervention was pilot-tested in secondary-level health care facilities where inpatient childbirth services are provided in both the maternity ward and labor room. In each district, we selected 3 public-sector health facilities (N=6) that provided at least standard basic emergency obstetric and newborn care services. Deliveries ranged from 40 to 300 per month at each facility.

### Study Population

Every woman who visited the study site for childbirth received the S-RMC intervention. The majority of women were admitted to the health facility, but a few were referred to other hospitals due to pregnancy complications. We only recruited women who gave birth at the health facility (study site) during the data collection period. Women were recruited at health facilities and interviewed at their homes for childbirth experiences at 42 days postpartum.

### Sample Size Calculation

Because this was a pilot study, calculations were performed using varying values of anticipated effect sizes. A sample of 308 was estimated to detect a statistical difference of 2.5 (10% reduction) in the mean composite score of mistreatment from 25 (a value taken from a previous study)^4^ with 90% power, 5% level of significance, 2 design effect, and 10% nonresponse/lost-to-follow-up at the home-based interview. The sample was deemed sufficient to perform additional analysis (e.g., estimating the prevalence of different types of mistreatment and examining the effect of the intervention on each type) while not negatively affecting study timelines. A total of 313 and 314 postnatal women were recruited to participate in pre- and post-intervention surveys, respectively.

### Sampling Strategy

We used a consecutive sampling technique for the recruitment of study participants. All women giving birth at the selected facilities during the data collection period were invited to participate in the survey until the desired sample was reached. The sample was proportionally distributed across health facilities according to the caseload of childbirths. Data collection was conducted simultaneously at all 6 facilities. Women who consented to both facility- and home-based interviews at 6 weeks postpartum were recruited for the survey. Participants who lived outside the study district or in hard-to-reach rural areas within the districts were excluded from the sample because of logistical constraints and potential security risks to data collectors. All participating women gave informed written consent.

### Study Instruments

We developed a comprehensive survey questionnaire comprising standard tools—with or without adaptation—that had been used previously in Pakistan. The questionnaire included questions on sociodemographics (adaptation of a Pakistan Demographic and Health Survey questionnaire),^19^ household poverty (using the progress-out-of-poverty tool),^20^ women’s involvement in household decision-making (standard inventory),^21^ functional disability (Washington Group’s 6 questions),^22^ health services (adapted from Pakistan 2017-2018 Demographic and Health Survey),^19^ screening of 42 days postpartum anxiety and depression (Patient Health Questionnaire – 4),^23^ and maternal satisfaction.^24^ To record experiences of mistreatment during childbirth, we adapted an open-access structured community survey instrument developed by an international group adopting an iterative mixed-method approach^24^ and used earlier in a multicountry study.^25^ The questionnaire provided information on the types of mistreatment identified in a systematic review.^2^ Additionally, we developed a 13-item perception-based tool to assess women’s experiences of “inclusive intrapartum care.” Women were asked if they thought they were treated unfairly by maternity staff because of their ethnic background, age, education, economic status, disability (6 conditions), mental condition, physical hygiene, or disease/illness. Responses were recorded on a scale from 0 (“strongly agree”) to 4 (“strongly disagree”).

### Data Collection and Management

In-person interviews were conducted by trained data collectors in privacy. Separate experienced data collection teams that were independent from the S-RMC intervention team were used for baseline and endline data collection. Women were recruited at health facilities at the time of discharge, and information on their sociodemographic characteristics was collected. Their experiences of childbirth were collected at 42 days postpartum at their homes. Data were collected electronically using tablets. The software was developed in Epicollect5 and had a built-in validation check to ensure data quality. Quality of data was routinely assessed by running simple frequencies and cross-classification analysis.

### Construction of Composite Variables

#### Outcome Variable

We used WHO’s mistreatment framework to guide construction of the overall composite measure of mistreatment and types of mistreatment. The overall composite score of mistreatment was constructed by adding the scores of 53 binary items, where “1” indicates “experience mistreatment,” and “0” indicates “not experienced.” The total raw score ranged from 0 to 53, which was linearly transformed on a scale of 0 to 100. The score indicates a cumulative level of mistreatment experienced by women during maternity care. A higher cumulative level of mistreatment indicates that women experienced a greater number of mistreatment manifestations. The same procedure was repeated for creating composite scores for each type of mistreatment, each ranging from 0 to 100. In addition to the continuous measures, we also created a binary measures for each type of mistreatment. Women who reported to have experienced at least 1 form of mistreatment of all possible manifestations in each given category were coded as “1” and “0” for those who experienced none. Details of individual items are in Supplement 1 Table S1.

#### Covariates

Based on our earlier study,^4^ we adjusted our main analysis for important covariates including age, primigravida (yes/no), native language (Sindhi/others), education (none/any formal), household poverty, women’s involvement in household decision-making, mode of birth (normal vaginal/cesarean), sought antenatal care for the index pregnancy (yes/no), and sex of index baby (boy/girl). Two composite indices were included as covariates—women’s involvement in household decision-making and household poverty. Because we used a validated tool, the poverty index was derived using the standard analytical approach as proposed by the Grameen Foundation.^20^ Similar to a methodology used previously,^21^ the composite measure of women’s decision-making was created by adding responses to 14 binary indicators (Supplement 1 Table S2), where “1” indicates women’s involvement in decision-making and “0” otherwise. A composite score ranged from 0 to 14, with a higher score reflecting a higher degree of decision-making autonomy.

### Statistical Analysis

Bivariate analyses employed independent t-test (continuous variables) and Pearson chi-square test (categorical variables) to determine whether significant differences existed in characteristics of participants at baseline and endline. We used 2 approaches to observe changes in women’s experiences of mistreatment: (1) mean difference in the continuous composite scores of overall mistreatment and types of mistreatment, which is indicative of the change in cumulative level mistreatment; and (2) proportion of women experiencing manifestations of mistreatment. In view of the type of outcomes variable (continuous or binary), multivariate linear and logistic regression models were used to estimate the differences in adjusted means and percentage, respectively. The models were adjusted for covariates, including age, order of pregnancy, ethnicity, education, household poverty, level of involvement in household decision-making, mode of birth, and sex of index baby (Table 1). Robust standard errors were estimated to account for the clustering effect of possible correlation of experiences among women from the same health facilities. Stata version 16.1 was used for all analyses, and *P* value of .05 was considered statistically significant. This article has been written in accordance with Revised Standards for Quality Improvement Reporting Excellence (SQUIRE 2.0) guidelines (Supplement 2).

**TABLE 1.** Components of S-RMC Intervention Package in Sindh, Pakistan

**Component**	**Actions**
Capacity-building	
Training of maternity teams on S-RMC	Labor room staff participate in 3-day S-RMC training course with modules on: Leadership and team-based maternity care Supportive and respectful maternity care Clarifying professional values to practice S-RMC Ethical, rights-based, and woman-centered care Psychosocial support and its implementation Operationalization of S-RMC strategyStudy team provides continuous supportive supervision by the study team (on average, 3 visits per health facility each month)
Improving governance and accountability mechanisms	
Front-end operationalization of S-RMC: Activities and/or processes that directly engage pregnant women and/or their companions at the maternity care facility	
Screening for vulnerabilities	Orient woman and companion about their rights and responsibilities and available support Screen for psychosocial vulnerabilities (e.g., anxiety, depression, and disability) and other sociodemographic vulnerabilities (e.g., poverty, lack of education or companionship, minority religion/caste)
Benchmarking respectful care and psychosocial support	Provide respectful care: avoiding violence, inclusive care, sharing information for informed decision-making, confidentiality, good rapport with woman, use of job aids Provide supportive care (i.e., psychosocial support): environmental support (cleanliness and privacy) and individual support (psychoeducation regarding needs and stressors, reducing stress, engaging companion, and promoting functioning)
Care coordination	For patient: share woman’s personalized preferences and needs with team members For staff: address care workers’ social and emotional needs
Assessing quality of care	Periodically assess woman’s experiences of S-RMC Implement complaint management system
Back-end operationalization of S-RMC: Managerial, administrative, and information-system activities and processes linked to the maternity team	
Enhanced management information system	Consolidate S-RMC-related data (e.g., vulnerability assessment and patient complaints and feedback)
Performance review and accountability	Conduct periodic (monthly) performance review meetings, including discussion of S-RMC performance and actions to be taken Identify S-RMC carer of the month
Continuous professional development	Provide on-the-job training and refreshers for staff by mental health first aider, a clinical member of the maternity team at each health facility

Abbreviation: S-RMC, supportive and respectful maternity care.

### Ethical Approval

The study protocol, the informed consent forms, and other appropriate documents were approved by the Ethics Review Committee of the Aga Khan University (Ref. ID: 2019-1683-5607) and Research Ethics Committee of the London School of Hygiene & Tropical Medicine (Ref. ID: 17886). The study has been registered with clinicaltrials.gov (registration number: NCT05146518).

## RESULTS

A total of 323 and 331 women were recruited at the health facilities at baseline and endline surveys, respectively. Of these women, 313 (96.9%) and 314 (94.9%) were successfully interviewed at their homes at baseline and endline, respectively. The primary reasons for nonresponse in both surveys were that women had migrated/shifted and or the data collection team was unable to locate the household. However, the refusal rate at the time of consent was less than 2% in both baseline and endline surveys.

We observed slight differences in sociodemographic characteristics of study participants between baseline and endline surveys. Although the majority of women across both the surveys were Sindhi, the proportion of Sindhi-speaking women was higher at endline. However, no significant differences were observed in reproductive health characteristics between baseline and endline samples. Most women were multigravida and had received antenatal care for the index childbirth. About 85% had a normal vaginal delivery, and around half the newborns were boys (Table 2).

**TABLE 2.** Sociodemographic and Reproductive Characteristics of Study Participants

	**Baseline, No. (%)** **(n=313)**	**Endline,** **No. (%)** **(n=314)**	***P* Value**
Age, mean (± SD)	28.0 (±6.4)	29.7 (±5.2)	<.001
Native language			.039
Sindhi	276 (88.2)	292 (93.0)	
Urdu, Punjabi, Balochi, Pushto, Saraiki, Brohi, Gujarati	37 (11.8)	22 (7.0)	
Women’s education			.693
None/illiterate	258 (82.4)	255 (81.2)	
Some formal education	55 (17.6)	59 (18.8)	
Poverty			.135
Women who live on less than US$1.25 a day	49.1 (±15.0)	50.9 (±15.2)	
Primigravida			.509
Yes	43 (13.7)	49 (15.6)	
No	270 (86.3)	265 (84.4)	
Received antenatal care for index pregnancy			.635
Yes	294 (93.9)	292 (93.0)	
No	19 (6.1)	22 (7.0)	
Mode of birth for current pregnancy			.286
Cesarean delivery	35 (11.2)	44 (14.0)	
Vaginal birth	278 (88.8)	270 (86.0)	
Sex of baby at most recent birth			.493
Boy	153 (48.9)	162 (51.6)	
Girl	159 (50.1)	152 (48.4)	
Twin (boy and girl)	1 (0.3)	0 (0)	

Table 3 presents changes in women’s overall experiences of S-RMC and its domains before and after the intervention. These changes are reported in terms of mean composite scores, which indicate the cumulative level of mistreatment. We observed a substantive reduction in the cumulative level of overall mistreatment from baseline (mean score 29.2) to endline (mean score 14.6), yielding a relative change of 50% (*P*<.001). Regarding the elements of respectful care, the highest relative change was noted in nonconfidential care (77.5%; *P*<.001), followed by physical abuse (75%; *P*<.001), verbal abuse (71.6%; *P*<.001), ineffective communication (60.1%; *P*<.001), health system culture and constraints (25.2%; *P*<.001), and lack of professional standards (17.3%; *P*<.001). The mean score for women’s perception of noninclusive care changed significantly from baseline (23.7) to endline (17.1). Notably, among women with 1 or more disability, the composite mean score for mistreatment reduced from 30.9 to 16.1 (relative change of 47.9%; *P*<.001). A relative change of 81.8% (*P*<.001) was also observed in the mean score for stigma and discrimination. Finally, for women’s experiences regarding lack of supportive care, the cumulative level changed significantly from 41.2 at baseline to 19.5 at endline, resulting in a relative change of 52.7% (*P*<.001). A substantial reduction (28.2%; *P*=.032) was observed in women’s experiences of anxiety and depressive symptoms at 42 days of postpartum before and after the intervention.

**TABLE 3.** Cumulative Level of Women’s Experiences of Supportive and Respectful Maternity Care^a^

**Characteristics**	**Baseline, Mean** **(SE) (n=313)**	**Endline, Mean** **(SE) (n=314)**	**Relative Change, %**	***P* Value**
Supportive and respectful maternity care (score range: 0–100)				
Composite mean score for mistreatment	29.2 (2.1)	14.6 (0.9)	50.0	<.001
Respectful care (score range: 0–100)				
Physical abuse	6.8 (0.7)	1.7 (0.7)	75.0	.001
Verbal abuse	10.2 (1.0)	2.9 (1.0)	71.6	.001
Sexual abuse	1.0 (0.2)	-	100.0	NC
Lack of professional standards	30.7 (2.0)	25.4 (1.2)	17.3	.06
Ineffective communication	38.3 (4.4)	15.3 (2.1)	60.1	.002
Nonconfidential care	62.6 (5.1)	14.1 (2.4)	77.5	<.001
Health system culture and constraints	69.4 (4.4)	51.9 (1.9)	25.2	.01
Inclusive care (score range: 0–100)				
Noninclusive care	23.7 (0.7)	17.1 (1.5)	27.8	.007
Stigma and discrimination	2.2 (0.7)	0.4 (0.1)	81.8	.044
Overall mean score for mistreatment among women with 1 or more disabilities^b^	30.8 (1.7)	16.3 (1.1)	47.1	<.001
Supportive care (score range: 0–100)				
Lack of supportive care	41.2 (3.9)	19.6 (2.5)	52.4	<.001
Postpartum anxiety and depression at 42 days, no. (%)	99 (32.2)	74 (23.1)	28.2	.032

Abbreviations: NC, not calculated; SE, standard error.

^a^ The mean scores at baseline and endline are estimated after adjusting for women’s age, primigravida, native language, education, household poverty, involvement in household decision-making, mode of birth, antenatal care for index birth, and sex of index baby.

^b^ Subsample: denominator includes only women with 1 or more disabilities (baseline: n=107 and endline: n=68).

We observed a substantive reduction in the cumulative level of overall mistreatment from baseline to endline.

Table 4 compares women’s overall experiences of mistreatment during facility-based births between baseline and endline (further details are in Supplement 1 Table S1). In the domain of respectful care, women’s reports of physical abuse reduced from 25.2% at baseline to 7.0% at endline (*P*<.001). This change was consistent across all manifestations of physical abuse, including fundal pressure, which was most common at baseline. Approximately 25% of women reported at least 1 form of verbal abuse at baseline, which reduced considerably to less than 10% at endline (*P*<.001), with a significant reduction in screaming, scolding, and threatening with poor outcomes. Although no significant change was observed in experience of lack of professional standards, specific changes were noted in the proportion of providers seeking permission before vaginal examinations (relative change 52.8%; *P*<.001). A relative change of 42.1% and 57.0% (*P*<.001) was observed in women’s experiences of ineffective communication and nonconfidentiality overall and as specific manifestations in each category. With respect to health system culture and constraints, we noted a substantial decrease in women’s reports of being forcefully asked for informal payment from 60.4% at baseline to 33.8% at endline.

**TABLE 4.** Prevalence of Women’s Experiences of Supportive and Respectful Maternity Care

**Characteristics**	**Baseline,** ^a^ **No. (%) (n=313)**	**Endline,** **No. (%) (n=314)**	**Relative Change, %**	***P* Value**
Respectful care				
Any physical abuse	79 (26.3)	22 (7.7)	70.7	.000
Slapped/punched	10 (3.5)	2 (0.7)	80.0	.039
Fundal pressure applied	62 (20.7)	19 (6.7)	67.6	<.001
Forced into woman’s nonpreferred birthing position	18 (5.9)	3 (1.1)	81.4	<.001
Verbal abuse	76 (24.6)	30 (9.4)	61.8	<.001
Screamed, shouted, or hissed at	50 (15.9)	12 (3.9)	75.5	<.001
Scolded	49 (15.7)	15 (4.8)	69.4	.000
Threatened with medical procedure, physical violence, poor outcome, or withholding care	43 (14.6)	10 (3.4)	76.7	<.001
Sexual abuse	10 (3.4)	0 (0)	100.0	NC
Stared at inappropriately by male staff	8 (2.7)	0 (0)	100.0	NC
Lack of professional standards	290 (92.0)	300 (95.3)	-3.3	.381
Abandoned for any reason during examination	86 (28.9)	66 (19.9)	31.1	.092
Permission not sought before vaginal examination	112 (36.5)	53 (16.5)	54.8	.002
Ineffective communication	257 (82.1)	149 (47.2)	42.1	<.001
No orientation about care processes at the facility	153 (49.0)	66 (20.8)	57.6	<.001
Failure to regularly share progress of labor with woman	107 (34.3)	30 (9.5)	72.3	<.001
No explanation offered of what to expect during labor and childbirth	170 (54.4)	77 (24.4)	54.9	<.001
Nonconfidential care	313 (100)	135 (43)	57.0	NC
No privacy for vaginal examinations	144 (46.9)	27 (8.3)	82.3	<.001
Health system culture and constraints	313 (100)	313 (99.7)	0.3	.318
Women forcefully asked for a bribe, informal payment, or gift for any reason	189 (61.0)	106 (33.0)	45.9	<.001
Women not told where to lodge a complaint	308 (98.5)	304 (96.6)	1.9	.289
Inclusive care				
Stigma and discrimination	32 (10.6)	6 (2.1)	80.2	<.001
Negative comment regarding economic circumstances	27 (8.8)	3 (1.1)	87.5	<.001
Overall mean score for mistreatment among women with 1 or more disability^b^	30.8 (1.7)	16.3 (1.1)	47.1	<.001
Supportive care				
Lack of supportive care	276 (88.5)	185 (58.0)	34.5	<.001
No encouragement/advice to breathe in and out to ease or hasten labor	131 (42.4)	72 (22.4)	47.2	<.001
No praise and reassurance when the woman abides by instructions	96 (31.2)	44 (13.7)	56.1	<.001
Companion not permitted to stay in labor room during childbirth	67 (22.2)	48 (14.5)	34.7	.048

Abbreviation: NC, not calculated.

a The percentages at baseline and endline are estimated after adjusting for woman’s age, primigravida, native language, education, household poverty, involvement in household decision-making, mode of birth, antenatal care for index birth, and sex of index baby.

b Denominator includes only women with 1 or more disability (baseline: n=107 and endline: n=68).

At baseline, nearly 10% of women received negative comments about their economic status, which reduced to 1.0% at endline (*P*<.001). Finally, women’s experiences of lack of supportive care reduced significantly from 88.2% at baseline to 58.2% at endline. This change was consistent across specific elements of supportive care (Supplement 1 includes analysis on all mistreatment items).

## DISCUSSION

Lack of S-RMC represents a complex health system challenge^26^ that negatively affects women’s labor and birthing experiences at health facilities. Our research empirically demonstrated that mistreatment of women during facility childbirth can be reduced by at least 50% and experience of childbirth considerably improved by enhancing inclusive care and psychosocial support. Effecting change requires addressing—systematically and simultaneously—complex dynamics at the nexus of pregnant women, service providers, and the health system.

### Perinatal Mental Health and Psychosocial Support

WHO’s mhGAP aims to implement strategies, evidence-based guidelines, and tools for establishing and delivering responsive mental health services for common mental health disorders in health care settings with limited resources or specialized services.^15^^,^^27^^,^^28^ The burden of poor mental health is onerous, and its implications—severe anxiety, depression, and psychosis linked with the maternity experience—are shocking. There is ample evidence from mhGAP-based interventions of perinatal depression in community settings (e.g., Thinking Healthy Program and group interpersonal psychotherapy).^29^^–^^33^ Within the health system, labor rooms and wards are the contact point for pregnant women, yet maternity teams are not technically equipped to support their clients’ perinatal mental health, especially their psychological and social needs.^9^^,^^10^ Moreover, maternity staff can suffer from stress and burnout due to their workload and need mental health support themselves.^34^ We believe our study is the first to use mhGAP in the maternity care setting.^14^ As a result of the implementation of S-RMC, the psychosocial support provided by maternity team members to birthing women resulted in a more than 50% reduction in women’s experiences of lack of supportive care, as well as a decrease in maternal postpartum anxiety and depressive symptoms.

As a result of the implementation of S-RMC, the psychosocial support provided by maternity team members to birthing women resulted in a more than 50% reduction in women’s experiences of lack of supportive care.

### Inclusive Care

Equality and inclusion within health care is a human right, enabling differences to be acknowledged and leading to individualized maternity care based on particular needs and beliefs. However, evidence of discriminatory, stigmatizing maternity care is widespread, including differential treatment by ethnicity, social or economic status, and reproductive and other personal characteristics.^4^^,^^35^ We have developed a research tool encompassing all the critical dimensions of inclusive care and have assessed change over time due to the intervention. Overall, women reported significant improvement in stigmatization and discrimination (i.e., more inclusive care) after the implementation of the S-RMC package.

### Respectful Care

Our intervention was different from other RMC packages (inclusive and psychosocially supportive care was central to ours), so a direct comparison of effectiveness was not possible. However, we found some consistent and contrasting results when compared with the existing literature. Our study’s 50% reduction in the cumulative level of mistreatment was consistent with another tested intervention in which a comparative before-and-after study showed a decrease of 66%.^36^ However, that research was implemented in just 1 tertiary-care hospital, and refusal rates ranged from 28% to 33%, whereas our study was implemented in 6 secondary-level hospitals. Also, the other intervention’s composite measure to assess mistreatment was based on only 14 questions covering the most common manifestations in contrast to our comprehensive 53-item mistreatment measure.^36^ We documented a relative reduction of 71% in the prevalence of physical abuse. Similar change was reported in other studies^7^; however, the range of baseline prevalence in those studies was less than 5%^7^ as opposed to 26% in ours. Again, the measurement of constructs used in other studies was based on a single question. We observed a marginal change in the category of professional standards (abandonment and nonconsented care), which is consistent with another trial that observed no clear difference or effect of intervention in opposite directions.^7^ The impact of earlier interventions on verbal abuse produced mixed findings, while we observed a significant relative reduction of 62%. Furthermore, earlier studies reported mixed evidence with respect to change in confidentiality or privacy,^7^ but we observed a reduction of 78%. In addition to conceptual and measurement differentials, earlier studies were part of broader health system intervention packages in which perspective and context are likely to have affected implementation and observed changes.

It is important to acknowledge that despite the broad spectrum and multicomponent design of the RMC intervention, while more general factors of health-system failure remain unchecked, its impact will have a certain threshold. Among these factors are organizational culture and oppressive hierarchies, poor infrastructure, incentives for quality, competence and care, and fair opportunities for career progression.

In recent decades, public health has made commendable gains in reducing the maternal mortality ratio worldwide, particularly in low- and middle-income countries.^37^ This reduction has been attributed to more women opting for institution-based deliveries. However, there have been negative consequences to this reduction. Whether it is because more women deliver at maternity facilities, their experiences have been better measured, or staff have worked under increased pressure with too few resources, the quality of care in such facilities has been woefully inadequate, and women have been routinely subjected to gross indifference, disrespect, and abuse.^2^ This has prompted much interventional research to improve the quality of maternity care in low-resource settings.^7^ However, the research has not packaged WHO’s complete recommendations for changes to health systems in low-resource settings to achieve a positive birthing experience.^7^ WHO’s guidance to a positive experience expands the conventional notion of RMC (i.e., absence of abuse) by including psychosocially supportive, inclusive, and respectful care.^6^ These aspects have been missing (unpublished data). Thus, RMC is a nascent, evolving phenomenon that needs evidence on contextualization, implementation, and assessment in low- and middle-income countries.^6^

Respectful and psychosocially supportive care is a core element of quality care—care beyond clinical safety and adeptness and based on social, psychological, and cultural competence.^5^^,^^6^ There is a lack of empirical, evidence-based research embedding RMC practices at the health facility and maternity team levels. Our research emphasizes the study methodology, implementation, and comparative evaluation of RMC.^8^ It pioneers the operationalization of WHO guidelines to provide empirical evidence for maternity policy and programming, capacity-building, research, and service evaluation to improve the birthing experience in low-resource settings.^38^

The S-RMC intervention rectifies potential sources of mistreatment through direct interaction with maternity care providers and staff (individual), the conditions of labor wards (structural), and the health system (process).^9^ The S-RMC resource package (handbook) is a vital element of the intervention. However, the intervention goes further by introducing changes under various health system blocks.^38^ Among the salient features of S-RMC intervention are the following changes in the health system perspective.

1. Having mechanisms in place to ensure that all women and their companions attending maternity facilities know their rights and responsibilities. As a part of dignified and respectful care, all women should receive psychosocial support. Inclusive care is provided by actively recognizing the differential care needs of women from underprivileged backgrounds.
2. Shifting the focus from individual behavior to the maternity team approach and care coordination.
3. Acknowledging the work strains of maternity staff and creating an internal support mechanism.
4. Fully integrating the package into health systems in low-resource settings, linking the front end (direct maternity care contacts) and back end (information, administrative, and professional development processes) of the health system to facilitate a positive birthing experience.
5. Strengthening in-built processes for regular learning from experiences and accountability in the event of mistreatment or inadequate service delivery.^38^

The ethos of coordinated and collaborative care by the maternity teams is a central component of the S-RMC intervention. Evidence acknowledges its importance and some practice models, but methods to measure quantitatively and determine the level of coordination among maternity staff are yet to be developed.^39^^,^^40^ Therefore, we explored this aspect qualitatively in the process evaluation. Maternity staff identified critical mechanisms of positive change in team coordination and collaborative care achieved through the S-RMC intervention. As a result, teams were better aware of patient needs and, therefore, collectively aimed to achieve personalized patient care. Nonclinical staff were also engaged in addressing psychosocial needs of pregnant women. In addition, unexpected improvements were noticed in handover practices during a shift change of the maternity team resulting in better continuity of services. Embedding processes of governance and accountability through periodic performance review based on facility-level data on S-RMC enabled them to make continuous improvements in care provision.

### Potential Implementation Challenges Regarding Scale-Up of S-RMC

A systematic review of RMC interventions affirms the pivotal role of leaders (policymakers, hospital managers, health system administrators, and health system experts) in scaling up the S-RMC intervention.^41^ Leaders must find the package acceptable. Scaling up requires significant efforts to build the capacity of service providers. Operationally, to sustain improvements in S-RMC, systemic changes (e.g., modifying management information systems and enhancing supervision and accountability mechanisms) would require endorsement from government leaders.

### Strengths and Limitations

The research has several distinguishing robust features, including that the study is underpinned by the theoretical frameworks of health systems, RMC, and behavior change.^8^ Psychosocial support during maternity care is based on successfully employing mhGAP—a well-established WHO task-shifting strategy for mental and social support in low-resource health system settings—for the first time in labor rooms and wards. Its effectiveness was assessed using psychometrically sound methods as part of multiple complementary qualitative and quantitative approaches to formulate, monitor, and evaluate the intervention.

A quasi-experimental study design with pre- and post-comparisons allowed the evaluation to change over time and contributed to fine-tuning the S-RMC package. However, a more formal effectiveness trial with a nonintervention comparison arm would help in embedding and scaling up within the health system. The prevalence of sexual abuse is generally marred by poor reporting in health system research. In our study, we cannot determine whether the low occurrence was valid or whether the abuse was underreported due to social or methodological constraints, although it is noteworthy that all clinical staff were female, while nonclinical staff were usually male and female. There is a case for developing more reliable measurements of sexual abuse in maternity and general care settings. Although more objective, direct observation of maternity staff/women interactions was used to inform the development of the intervention, we did not include this in our evaluation approaches to avoid contaminating our findings with the Hawthorne effect (i.e., maternity staff have received training in S-RMC).

Global evidence of all types of mistreatment, discrimination, and stigmatization, as well as an absence of supportive care during facility childbirth, is overwhelming.^2^ In meeting this challenge, the S-RMC implementation has shown great promise and instituted solutions, but further research is needed to hone its approach and deliver it in diverse contexts. In addition, current patient satisfaction measuring tools and approaches lack conceptual depth and methodological finesse. Patients’ satisfaction with maternity services is another aspect of RMC to explore in future health system research. The study was conducted at secondary health care facilities that offer basic emergency obstetric and newborn care because this is the first level of the local health system where formal labor room and ward facilities are offered. However, to increase the generalizability of the S-RMC package, its effectiveness needs to be established at the primary and tertiary health care levels of the health system. Finally, replicating S-RMC’s principles in other health care services (e.g., antenatal and postpartum care, including newborn care and family planning) could be a useful direction for future research.

## CONCLUSION

A systematic approach can effectively address critical gaps in the implementation of S-RMC. Health providers are more likely to bring change when adequately supported by health-system processes, learning opportunities, and better-oriented patients. Interventions to improve S-RMC can be successful if underpinned by an in-depth understanding of behavior change and of health-system drivers and gaps. Then, they can systematically focus on the calibration of health system blocks from the front end and back end of the system to enable positive birthing experiences, thus improving the quality of maternity care services in low-resource settings.

## Supplementary Material

GHSP-D-22-00513-supplement.pdf

## Notes

**Peer Reviewed**

**First published online:** June 19, 2023.
