# Supplementary material for: Promoting Supportive and Respectful Maternity Care in Public Health Facilities in Sindh, Pakistan: A Theory-Informed Health System Intervention
Source: Glob Health Sci Pract. 2023 Jun 21;11(3):e2200513. doi: 10.9745/GHSP-D-22-00513 (PMC10285721; doi:10.9745/GHSP-D-22-00513)
Supplement: GHSP-D-22-00513-supplement.pdf [file GHSP-D-22-00513-supplement.pdf]

Supplement to: Avan BI, Hameed W, Khan B, Asim M, Saleem S, Siddiqi S. Promoting supportive and respectful maternity care in public health facilities in Sindh, Pakistan: a theory-informed health system intervention. *Glob Health Sci Pract*. 2023;11(3):e2200513. https://doi.org/10.9745/GHSP-D-22-00513

SUPPLEMENT 1: Additional analysis

Table S1: Women's experiences of Supportive and Dignified Maternity Care – crude difference between baseline and endline assessments

Characteristics	Baseline n = 313		Endline n = 314		Relative change	p - value
	n	%	n	%	%	
**I. Respectful Care**						
**Composite mean percentage score for mistreatment (Range: 0-100)**						
Means (± SD) (Range: 0-100)	29.1	12.3	14.7	7.6	49.5	<0.001
**Any physical abuse**	**79**	**25.2**	**22**	**7.0**	72.2	**<0.001**
Slapped/punched	10	3.2	2	0.6	80.0	0.019
Fundal pressure applied	62	19.8	19	6.1	69.4	<0.001
Forced into woman's non-preferred birthing position	18	5.8	3	1	83.3	0.001
Pinched irritably	14	4.5	1	0.3	92.9	0.001
Hit with an instrument/gag	10	3.2	2	0.6	80.0	0.019
Tied or held down	18	5.8	2	0.6	88.9	<0.001
**Intensity of physical abuse**						
**0**	**234**	**74.8**	**292**	**93.0**	-24.8	
1	53	16.9	18	5.7	66.0	
2	11	3.5	2	.6	81.8	
3	7	2.2	1	.3	85.7	
4	5	1.6	1	.3	80.0	
5	2	.6	0	.0	100.0	
6	1	.3	0	.0	100.0	
Actual raw score: range 0-6 (mean and SD)	0.4	0.9	0.1	0.4	75.0	<0.001
Percentage score: range 0-100 (mean and SD)	7.0	15.5	1.5	6.6	78.6	<0.001
**Any verbal abuse**	**76**	**24.3**	**30**	**9.6**	60.5	**<0.001**
Screamed, shouted or hissed at	50	16.0	12	3.8	76.0	<0.001
Scolded	49	15.7	15	4.8	69.4	<0.001
Threatened with medical procedure, physical violence, poor outcome, or withholding care	43	13.7	10	3.2	76.7	<0.001
Insulted/mocked	26	8.3	6	1.9	76.9	<0.001

Characteristics	Baseline n = 313		Endline n = 314		Relative change	p - value
	n	%	n	%	%	
Passed negative comments about physical appearance of woman or baby, or about sexual activity	17	5.4	8	2.5	52.9	0.065
Blamed for something that happened to woman or baby	8	2.6	2	0.6	75.0	0.055
**Intensity of verbal abuse score**						
0	237	75.7	284	90.4	-19.8	
1	27	8.6	15	4.8	44.4	
2	15	4.8	9	2.9	40.0	
3	12	3.8	4	1.3	66.7	
4	13	4.2	2	.6	84.6	
5	6	1.9	0	.0	100.0	
6	3	1.0	0	.0	100.0	
Actual raw score: range 0-6 (mean and SD)	0.6	1.3	0.2	0.6	66.7	<0.001
Percentage score: range 0-100 (mean and SD)	10.3	22.0	2.8	9.9	72.8	<0.001
**Sexual abuse**	**10**	**3.2**	**0**	**0**	100.0	
Stared at inappropriately by male staff	8	2.6	0	0	100.0	0.004
Said things of a sexual nature about physical appearance	4	1.3	0	0	100.0	0.044
Lewd songs, lascivious sounds, immoral talk, jokes, stories of a sexual nature	4	1.3	0	0	100.0	0.044
Misconducted (e.g., licking own lips, touching own private body parts; hinting inappropriately, showing pornographic/nude photograph or video	2	0.6	0	0	100.0	0.156
Touched private body parts (vagina, breast, thigh, buttock, lips) in a sexual manner	0	0	0	0	-	NC
Threatened, or forced to engage in, a sexual activity	1	0.3	0	0	100.0	0.316
Raped	0	0	0	0	-	NC
**Intensity of sexual abuse score**						
0	303	96.8	314	100.0	-3.6	
1	7	2.2	0	.0	100.0	
3	1	.3	0	.0	100.0	
4	1	.3	0	.0	100.0	

Characteristics	Baseline n = 313		Endline n = 314		Relative change	p - value
	n	%	n	%	%	
5	1	.3	0	.0	100.0	
mean (SD)	0.1	0.4	00	00	100.0	
Actual raw score: range 0-6 (mean and SD)	0.1	0.4	0	0	100.0	0.012
Percentage score: range 0-100 (mean and SD)	0.9	6.0	0	0	100.0	0.012
**Lack of inclusive care/stigma and discrimination**	**32**	**10.2**	**6**	**1.9**	81.3	
Negative comment regarding economic circumstances	27	8.6	3	1	88.9	0.000
Negative comment regarding ethnicity, race, tribe or culture	3	1	1	0.3	66.7	0.373
Negative comment regarding religion	2	0.6	0	0	100.0	0.249
Negative comment regarding age	7	2.2	3	1	57.1	0.200
Negative comment regarding psychological distress	4	1.3	0	0	100.0	0.062
Negative comment regarding education or literacy	4	1.3	1	0.3	75.0	0.217
Negative comment regarding disability or physical disease	2	0.6	0	0	100.0	0.249
**Intensity of stigma and discrimination score**						
**0**	**281**	**89.8**	**308**	**98.1**	-9.6	
1	22	7.0	5	1.6	77.3	
2	5	1.6	0	.0	100.0	
3	3	1.0	1	.3	66.7	
4	2	.6	0	.0	100.0	
Actual raw score: range 0-7 (mean and SD)	0.2	0.6	0.03	0.2	85.0	<0.001
Percentage score: range 0-100 (mean and SD)	2.2	7.8	0.4	3.0	81.8	<0.001
**Non-confidential care**	**313**	**100**	**135**	**43**	56.9	
No privacy for vaginal examinations	120	44.3	26	8.8	78.3	0.000
Woman not covered while taken to and from birthing room	310	99.9	17	5.4	94.5	0.000
No curtain to maintain privacy from other patients, family members or staff	170	54.3	32	10.2	81.2	0.000
Discussion of personal information/diagnosis about health not conducted privately	157	50.2	104	33.1	33.8	0.000

Characteristics	Baseline n = 313		Endline n = 314		Relative change	p - value
	n	%	n	%	%	
**Intensity of confidential care score**						
0	0	0.0	179	57.0	-	
1	81	25.9	98	31.2	-21.0	
2	92	29.4	30	9.6	67.4	
3	68	21.7	7	2.2	89.7	
4	72	23.0	0	.0	100.0	
Actual raw score: range 0-7 (mean and SD)	2.5	1.2	0.6	0.8	76.0	<0.001
Percentage score: range 0-100 (mean and SD)	62.4	28.7	14.3	19.1	77.1	<0.001
**Ineffective communication**	**257**	**82.1**	**149**	**47.5**	42.0	
No orientation about care processes at the hospital	153	48.9	66	21	56.9	0.000
Failure to regularly share progress of labour with woman	107	34.2	30	9.6	72.0	0.000
No explanation offered of what to expect during labour and childbirth	170	54.3	77	24.5	54.7	0.000
Not providing information in easy and understandable manner	62	19.8	10	3.2	83.9	0.000
Not sharing diagnosis/results of examination with woman	63	20.1	23	7.3	63.5	0.000
Not informing woman about toilet facilities upon admission	164	52.4	83	26.4	49.4	0.000
**Intensity of Ineffective communication**						
0	56	17.9	165	52.5	-194.6	
1	63	20.1	78	24.8	-23.8	
2	62	19.8	27	8.6	56.5	
3	46	14.7	29	9.2	37.0	
4	47	15.0	7	2.2	85.1	
5	28	8.9	6	1.9	78.6	
6	11	3.5	2	.6	81.8	
Actual raw score: range 0-6 (mean and SD)	2.3	1.7	0.9	1.3	60.9	<0.001
Percentage score: range 0-100 (mean and SD)	38.3	28.5	15.3	21.3	60.1	<0.001
**Lack of supportive care**	**276**	**88.2**	**185**	**58.9**	33.0	

Supplement to: Avan BI, Hameed W, Khan B, Asim M, Saleem S, Siddiqi S. Promoting supportive and respectful maternity care in public health facilities in Sindh, Pakistan: a theory-informed health system intervention. *Glob Health Sci Pract*. 2023;11(3):e2200513. https://doi.org/10.9745/GHSP-D-22-00513

Characteristics	Baseline n = 313		Endline n = 314		Relative change	p - value
	n	%	n	%	%	
No encouragement/advice to breathe in and out to ease or hasten labour	131	41.9	72	22.9	45.0	<0.001
No praise and reassurance when the woman abides by instructions	96	30.7	44	14	54.2	<0.001
Companion not permitted to stay in labour room during childbirth	67	21.4	48	15.3	28.4	0.048
No encouragement, or clarification of doubts and ambiguities regarding delivery	139	44.4	68	21.7	51.1	<0.001
No encouragement of woman to express feeling (e.g., fear, anger, stress, fatigue, pain)	102	32.6	38	12.1	62.7	0.000
No counselling offered to woman in an effective manner to overcome her fear	107	34.2	87	27.3	18.7	0.079
No assistance offered to woman to ambulate during labour when needed	250	79.9	83	26.4	66.8	<0.001
**Intensity of lack of supportive care**						
0	37	11.8	129	41.1	-248.6	
1	81	25.9	70	22.3	13.6	
2	59	18.8	46	14.6	22.0	
3	23	7.3	30	9.6	-30.4	
4	28	8.9	14	4.5	50.0	
5	18	5.8	19	6.1	-5.6	
6	47	15.0	5	1.6	89.4	
7	20	6.4	1	.3	95.0	
Actual raw score: range 0-7 (mean and SD)	2.9	2.2	1.4	1.6	51.7	<0.001
Percentage score: range 0-100 (mean and SD)	40.7	31.9	20.0	23.3	50.9	<0.001
**Lack of professional standards**	**290**	**92.7**	**300**	**95.5**	-3.4	
Abandoned for any reason during examination	86	27.5	66	21.0	23.3	0.059
Permission not sought before vaginal examination	112	35.8	53	16.9	52.7	0.000
Detained woman or child for any reason	3	1.0	6	1.9	-100.0	0.505
Not allowed woman to walk or move around during labour with no reason given	43	13.7	25	8.0	41.9	0.020
Staff interrupted woman when speaking or asking them to do something	83	26.5	52	16.6	37.3	0.002

Supplement to: Avan BI, Hameed W, Khan B, Asim M, Saleem S, Siddiqi S. Promoting supportive and respectful maternity care in public health facilities in Sindh, Pakistan: a theory-informed health system intervention. *Glob Health Sci Pract*. 2023;11(3):e2200513. https://doi.org/10.9745/GHSP-D-22-00513

Characteristics	Baseline n = 313		Endline n = 314		Relative change	p - value
	n	%	n	%	%	
**Intensity of professional standards score**						
0	23	7.3	14	4.5	39.1	
1	115	36.7	169	53.8	-47.0	
2	100	31.9	87	27.7	13.0	
3	49	15.7	35	11.1	28.6	
4	22	7.0	7	2.2	68.2	
5	4	1.3	2	.6	50.0	
Actual raw score: range 0-6 (mean and SD)	1.8	1.1	1.6	0.9	11.1	0.001
Percentage score: range 0-100 (mean and SD)	30.4	18.2	25.8	14.7	15.1	0.001
**Health system constraints and conditions**	**313**	**100**	**313**	**99.7**	0.0	
Woman forcefully asked for a bribe, informal payment or gift for any reason	189	60.4	106	33.8	43.9	0.000
Women not told where to lodge a complaint	308	98.4	304	96.8	1.3	0.193
Non-useable toilet	107	34.2	28	8.9	73.8	<0.001
Bed linen were in poor condition, unclean, or missing	206	65.8	126	40.1	38.8	
No or inadequate seating for companion in either ward or labour room	279	89.1	248	79.0	11.1	0.310
**Intensity of health system condition**						
0	0	.0	1	.3		
1	6	1.9	25	8.0	-316.7	
2	35	11.2	121	38.5	-245.7	
3	131	41.9	128	40.8	2.3	
4	85	27.2	34	10.8	60.0	
5	56	17.9	5	1.6	91.1	
Actual raw score: range 0-5 (mean and SD)	3.5	1.0	2.6	0.9	25.7	<0.001
Percentage score: range 0-100 (mean and SD)	69.6	19.5	51.7	17.2	25.7	<0.001

Note: The grey highlighted row are also presented in the main manuscript tables

Table S2: Consensual care for surgical procedures – a sub-group analysis

Characteristics	Baseline N = 313		Endline N = 314		Relative change	p - value
	n	%	n	%	%	
**Non-consented care**						
Underwent C-section (inclusive of both elective or emergency)	35	11.2	44	14	-25.0	0.286
Didn't seek informed consent for C-section	8	22.9	12	27.3	-23.0	0.604
Underwent episiotomy	30	9.6	30	9.6	0.0	0.990
Didn't seek informed consent for episiotomy procedure	19	63.3	14	46.7	26.2	0.194
Underwent augmentation of labour procedure	158	50.5	202	64.3	-27.3	0.000
Didn't seek informed consent for augmentation of labour procedure	55	34.8	61	30.2	13.2	0.353

Table S2 presents the sub-group analysis on consensual care for different maternity care procedures. The denominator for consented care for each procedure was women who had undergone that procedure.

Supplement to: Avan BI, Hameed W, Khan B, Asim M, Saleem S, Siddiqi S. Promoting supportive and respectful maternity care in public health facilities in Sindh, Pakistan: a theory-informed health system intervention. *Glob Health Sci Pract*. 2023;11(3):e2200513. https://doi.org/10.9745/GHSP-D-22-00513

SUPPLEMENT 2. Revised Standards for Quality Improvement Reporting Excellence (SQUIRE 2.0) September 15, 2015

Text Section and Item Nam		Section or Item description
**Title and abstract**		
1	Title	Yes – patient-centeredness and equity of healthcare
2	Abstract	Yes, all key terms are used
**Introduction**		
3	Problem description	Page 3
4	Available knowledge	Page 3
5	Rationale	Page 3
6	Specific aims	Page 3
**Methods**		
7	Context	Page 5
8	Intervention	Page 4 & 5
9	Study of the intervention	Page 5
10	Measures	Page 6 and 7
11	Analysis	Page 7
12	Ethical consideration	Page 5
**Results**		
13	Results	Page 7 to 10
**Discussion**		
14	Summary	Page 11
15	Interpretation	Page 11, 12 & 13
16	Limitations	Page 13
17	Conclusion	Page 13 & 14
**Other information**		
18	Funding	Yes, mentioned in the application
